# Case report: A case of bradycardia triggered by diarrhea

**DOI:** 10.3389/fmed.2024.1405494

**Published:** 2024-05-30

**Authors:** Meixian Lei, Yuan Cao, Mingqing Yuan, Jie Xiong, Huabin He

**Affiliations:** Department of Cardiology, Jiujiang First People’s Hospital, Jiujiang, China

**Keywords:** hyperkalemia, bradycardia, renal failure, shock, diarrhea

## Abstract

BRASH syndrome is a vicious cycle of hyperkalemia and bradycardia and is an under-recognized life-threatening clinical diagnosis. It is usually initiated by hypovolemia or hyperkalemia. We report here on the case of a 92-year-old man with hypertension and heart failure who presented to the emergency department with weakness following diarrhea. He was on amlodipine, benazepril, metoprolol, furosemide and spironolactone. The patient’s blood pressure was 88/53 mmHg and the serum creatinine was 241 μmol/L. Within 2 h, the patient’s heart rate decreased from 58 beats per minute to 26 beats per minute, and serum potassium levels gradually increased from 6.07 mmol/L to 7.3 mmol/L. The electrocardiogram showed a junctional escape rhythm with accidental sinus capture. The diagnosis of BRASH syndrome was made based on clinical symptoms, a biochemical profile and the results of an electrocardiogram. The patient was rapidly stabilized with the administration of intravenous calcium gluconate, dextrose and insulin, 5% sodium bicarbonate, 0.9% sodium chloride, furosemide, and oral zirconium cyclosilicate. Sinus rhythm at a heart rate of 75 bpm was detected 5 h later, along with normal serum potassium levels. After 2 weeks, kidney function returned to normal. Clinicians should be alert to patients with hyperkalemia and maintain a high index of suspicion for BRASH syndrome. Timely diagnosis and comprehensive intervention are critical for better outcomes in managing patients with BRASH.

## Introduction

The BRASH (bradycardia, renal failure, atrioventricular node blockers, shock, and hyperkalemia) syndrome is characterized by a vicious cycle of bradycardia resulting from hyperkalemia (1). Hyperkalemia is an anticipated complication in patients with renal failure, but it may also occur in patients without a history of renal disease. Diarrhea is a rare cause of hyperkalemia, particularly among patients without renal insufficiency. Thus, we present a case of BRASH syndrome that was caused by diarrhea in a hypertensive patient, a combination that has never occurred before.

## Case report

A 92-year-old man was admitted to the hospital due to sudden, generalized weakness. Six hours prior to admission, the patient had diarrhea without nausea or vomiting. He felt a progressive fatigue in his limbs. He did not report symptoms such as amaurosis, syncope, or somatosensory disturbance. He has hypertension, heart failure with preserved ejection fraction, and pleural effusion in his past, but he does not have chronic kidney disease. The patient was prescribed daily doses of amlodipine 5 mg, benazepril 10 mg, metoprolol 12.5 mg twice daily, furosemide 20 mg, and spironolactone 20 mg.

**Figure 1:**
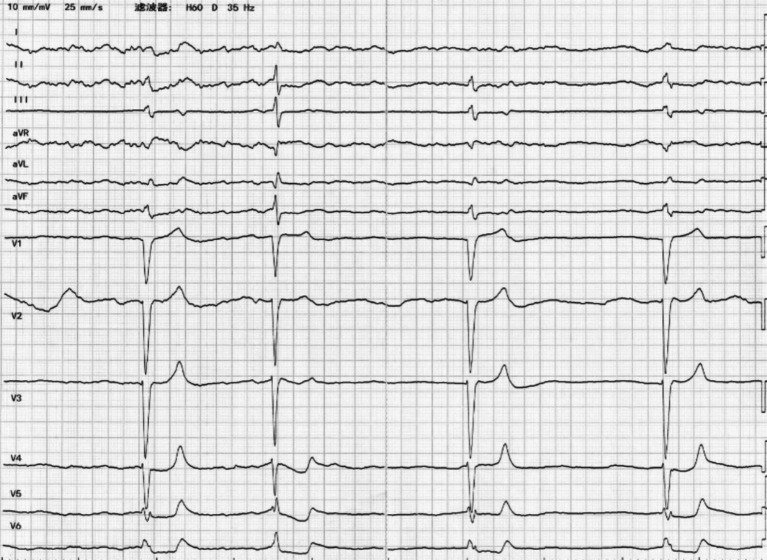
The ECG on admission revealed sinus arrest, junctional escape rhythm with accidental sinus capture, QRS prolongation, and tall, peaked T waves.

The physician initially assessed the patient, recorded bradycardia (54 beats per minute[bpm]) and hypotension (88/53 mmHg), and proceeded with a computed tomography (CT) scan of the head and serum electrolyte testing. A CT scan of the skull showed normal results. Laboratory tests showed hyperkalemia, hyponatremia, hypochloremia, anion gap metabolic acidosis, and renal dysfunction (Table 1, Day 0, 0 Hour) 2 hours later. A repeat serum electrolyte test was performed to evaluate for pseudohyperkalemia. During the re-examination, the patient exhibited a pulse rate of 26 bpm, blood pressure of 86/50 mmHg and room air oxygen saturation of 96%. The patient’s heart rate was found to be slow and irregular, without any murmur. His abdomen was flat and soft, with no sign of tenderness or rebound tenderness. The extremities were negative for edema, were symmetrical with a 3/5 strength, and were asymptomatic of neurological disorders. Laboratory tests showed a progressive increase in serum potassium (Table 1, Day 0, 2 Hours). An electrocardiogram showed sinus arrest, junctional escape rhythm with accidental sinus capture, left bundle branch block (QRS duration of 136 ms), and hyperacute tented T waves (Figure 1).

**Table 1:** Laboratory results from clinical courses.

Blood analytes	Day 0			Day 1	Day 3	Day 14	Normal range
	0 Hour	2 Hours	5 Hours				
Sodium (mmol/L)	124	120	123	125.8	132.8	140.8	135–147
Potassium (mmol/L)	6.07	7.3	5.32	5.15	3.79	4.15	3.5–5.5
Chlorine	87	86	87	87.1	92.4	108.9	96–112
Anion gap (mmol/L)	18.3	21.6	16.6	13.6	14.2	10.2	8–16
Urea (mmol/L)	18.26			18.31	10.57	4.1	1.70–8.30
Creatinine (μmol/L)	241			237	176	100	59–104

**Figure 2:**
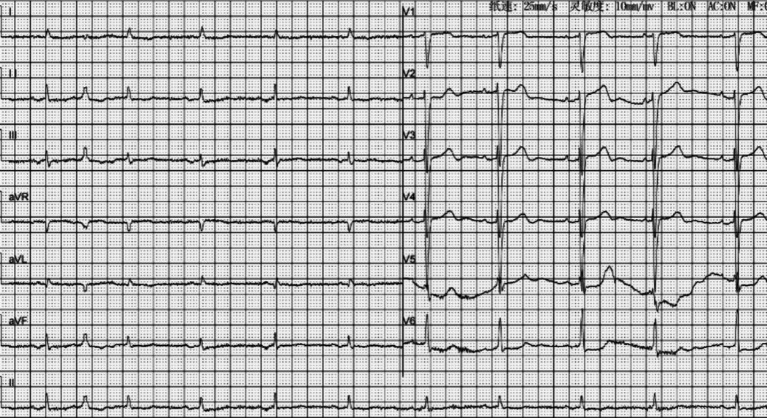
The post-treatment ECG showed sinus rhythm with ventricular premature beat.

In addition to the discontinuation of amlodipine, benazepril, metoprolol, and spironolactone, the patient was administrated of intravenously calcium gluconate, dextrose and insulin, 5% sodium bicarbonate, 0.9% sodium chloride, furosemide, and oral zirconium cyclosilicate. Five hours later, Sinus rhythm was recorded by the electrocardiographic monitoring with a heart rate of 75 bpm, and serum potassium levels were with normal range (Table 1, Day 0, 5 Hours). The anion gap was within the normal limits on day 1 (Table 1, Day 1). Treatment continued with 0.9% sodium chloride until the serum sodium and chlorine levels approached normal (Table 1, Day 3). the ECG was re-examination to reveal a sinus rhythm with a normal QRS duration and a ventricular premature beat (Figure 2). After the symptoms were resolved, the patient was discharged. He took amlodipine 5 mg daily and stop taking benazepril, metoprolol, furosemide and spironolactone. His kidney function returned to normal after 2 weeks (Table 1, Day 14).

## Discussion

Potassium is the most abundant cation in the human body, with extracellular fluid accounting for only 2% (3.5–5.0 mmoL/L) (2). The level of potassium in serum remains within a relatively narrow normal range. A complex interplay of regulatory mechanisms is needed to maintain normal potassium balance, which involves the transfer of potassium between the extracellular and intracellular, and the regulation of potassium excretion in the kidney. Hyperkalemia is a cause of heart block, arrhythmia, syncope, and cardiac arrest. It can be precipitated by multiple factors, including increased intake, decreased renal excretion, or a shift of K^+^ from cells to extracellular fluid, with the latter two being common causes (2).

The clinical syndrome of acute kidney injury (AKI) is characterized by a rapid decrease in renal function. Prerenal factors are the most common factors that lead to this decline. The cause of these factors is insufficient renal perfusion, with a deficiency in both absolute blood volume and relative volume due to vasodilation. In hospitalized adult patients with diarrhea, 10% will develop AKI, with the highest incidence occurring in the elderly (3). Chronic kidney disease (CKD) and hypertension increase the risk of AKI in patients with diarrhea, possibly due to decreased renal reserve, the exacerbating effects of diuretics and inhibitors of the renin-angiotensin-aldosterone antagonists.

**Figure 3:**
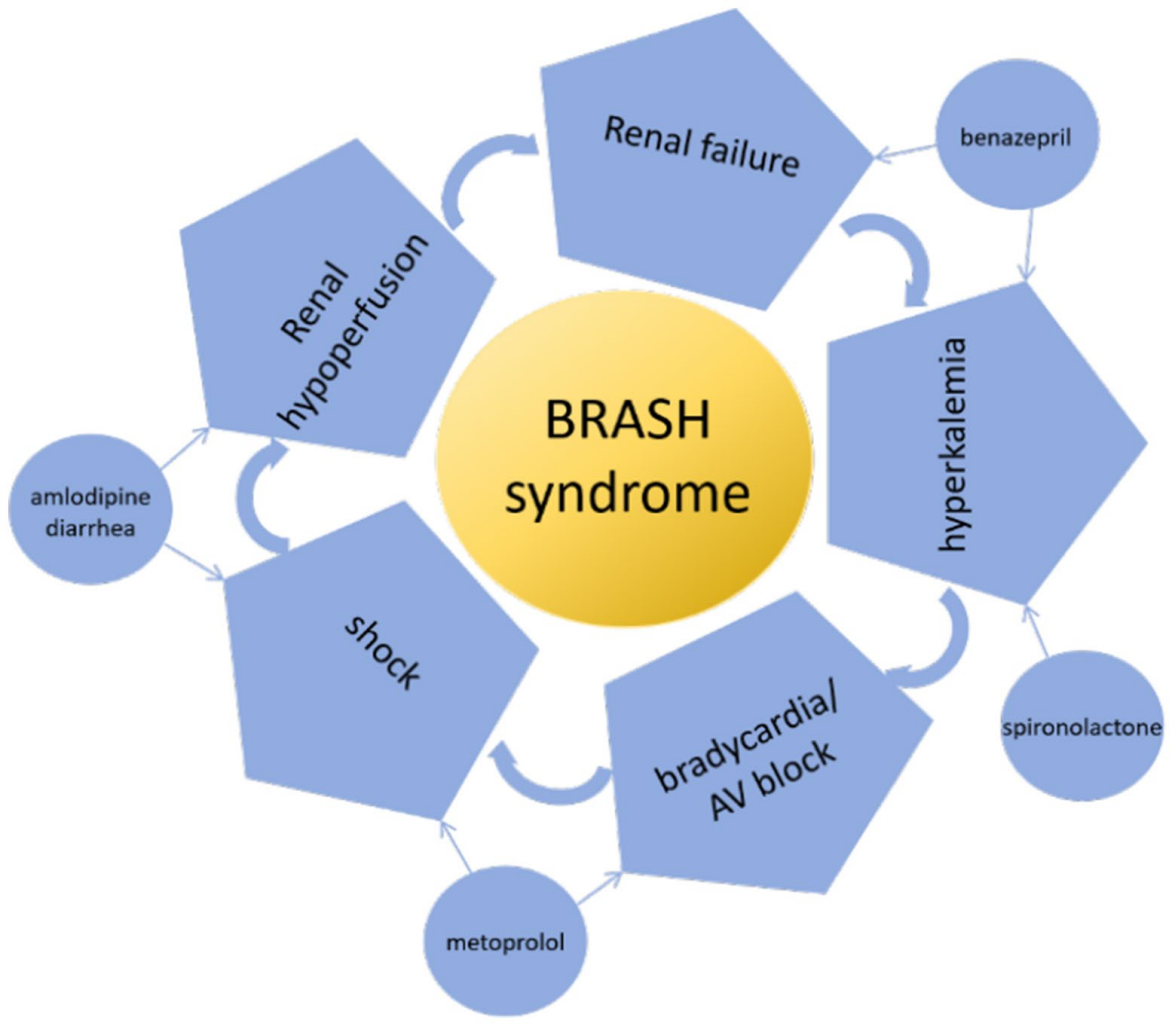
Pathophysiologic mechanisms of BRASH syndrome.

When evaluating hyperkalemia, pseudohyperkalemia should be excluded first in patients without risk factors. In this case, the patient had diarrhea and was taking furosemide and antihypertensive medications, resulting in hypovolemia. This resulted in a decreased renal perfusion and the onset of AKI. Benazepril and spironolactone combined to worsen renal potassium excretion when AKI was present. Diarrhea-induced metabolic acidosis resulted in potassium shifting from the cells to the extracellular fluid. Decreased renal potassium excretion and increased extracellular shift result in the occurrence of hyperkalemia, ultimately leading to BRASH syndrome (Figure 3).

Since the introduction of the BRASH syndrome concept (4) and the publication of case reports (5, 6), BRASH syndrome has been identified (1) as a vicious circle of hyperkalemia resulting in bradycardia (4). Afterwards, bradycardia results in decreased cardiac output, shock, poor tissue perfusion, renal failure, and an aggravation of hyperkalemia and the deterioration of bradycardia. BRASH syndrome can be caused by both hyperkalemia and hypovolemia, such as hypovolemia caused by anaphylaxis (5). It is more prevalent among the elderly, especially when considering polypharmacy and comorbidities (6–8). Hypertension is one of the most common co-morbidities (9, 10). BRASH syndrome can be exacerbated by taking medications that reduce atrioventricular node conduction and renin-angiotensin-aldosterone inhibitors (5, 9, 10). The presence of these risk factors in our patient, including advanced age, concomitant hypertension, β-blockers use, and ACEI utilization, may potentially precipitate the onset of BRASH syndrome.

BRASH syndrome is a life-threatening clinical diagnosis that is often under-recognized. The non-specific clinical symptoms of BRASH syndrome, including fatigue, muscle weakness, paresthesia, dizziness, and syncope, can be caused by multiple factors and can present significant challenges for clinical management. Therefore, a comprehensive and meticulous medical collection of medical history, in addition to auxiliary examinations, is often crucial (11). Early recognition of BRASH syndrome can lead to favorable outcomes for patients, but a neglected cases can result in severe consequences, including death, without prompt attention (11–13). It is important to consider multiple management strategies based on the pathophysiology of BRASH syndrome, rather than focusing solely on a single electrolyte disturbance or relying on ‘Classic’ advanced cardiac life support algorithms (11). The coordinated management strategy of multiple non-invasive therapies involves discontinuing triggering drugs and shifting and eliminating potassium (e.g., sodium bicarbonate, insulin/glucose, nebulized albuterol, loop diuretics), stabilizing myocardial membrane potential (calcium gluconate), using catecholamines (isoproterenol, epinephrine) (4), and fluid resuscitation if hypovolemia. This approach has the potential to prevent more invasive treatments, such as transvenous pacing and hemodialysis (4).

## Conclusion

BRASH syndrome is a vicious cycle of hyperkalemia and bradycardia, and is largely under-recognized as a life-threatening clinical diagnosis. Hyperkalemia can be a potential complication for patients with renal failure. Although hypokalemia is common in patients with diarrhea, approximately 10% of diarrhea patients will progress to AKI, which may ultimately lead to hyperkalemia. When elderly patients, particularly those with comorbidities such as CKD and hypertension, are experiencing diarrhea symptoms, clinicians should keep track of their serum potassium and kidney function. It is important to be alert for patients with hyperkalemia and maintain a high index of suspicion for BRASH syndrome. To achieve better outcomes in managing patients with BRASH, timely diagnosis and comprehensive intervention are vital.

## Data availability statement

The original contributions presented in the study are included in the article/supplementary material, further inquiries can be directed to the corresponding authors.

## Ethics statement

Written informed consent was obtained from the individual(s) for the publication of any potentially identifiable images or data included in this article.

## Author contributions

ML: Writing – original draft, Writing – review & editing. YC: Data curation, Writing – original draft. MY: Data curation, Writing – original draft. JX: Data curation, Writing – original draft. HH: Data curation, Validation, Writing – review & editing.
